# Rescuers at Risk: Posttraumatic Stress Symptoms Among Police Officers, Fire Fighters, Ambulance Personnel, and Emergency and Psychiatric Nurses

**DOI:** 10.3389/fpsyt.2020.602064

**Published:** 2021-01-19

**Authors:** Leila M. Soravia, Simon Schwab, Sebastian Walther, Thomas Müller

**Affiliations:** ^1^Translational Research Center, University Hospital of Psychiatry, University of Bern, Bern, Switzerland; ^2^Center for Reproducible Science (CRS), University of Zurich, Zurich, Switzerland; ^3^Epidemiology, Biostatistics and Prevention Institute (EPBI), University of Zurich, Zurich, Switzerland; ^4^Private Clinic Meiringen, Meiringen, Switzerland

**Keywords:** PTSD, coping, risk factors, rescue workers, well-being

## Abstract

Emergency personnel and rescue workers may be at a risk of posttraumatic stress symptoms (PTSS) due to exposure to trauma and work-related stressors. Though rescuers of different professions are often engaged in the same type of emergency, they have different tasks and responsibilities and receive different training in coping with traumatic events and stress; hence, we speculated that the salience of identified risk factors for PTSS vary across their respective professions. The present cross-sectional survey aimed to identify influencing variables on PTSS, well-being, and suicidal ideation that can act differently across professions of rescue workers and emergency personnel. In this anonymous online study, data from 1,002 rescue workers and emergency personnel in Switzerland, were collected: 499 police officers, 239 firefighters, 97 ambulance personnel, and 85 emergency and 82 psychiatric nurses. PTSS, coping strategies, well-being, suicidal ideation, previously experienced and work-related trauma, and self-efficacy were measured and analyzed using multiple regression and structural equation modeling (SEM). The prevalence of suspected posttraumatic stress disorder varied across the professions, ranged from 8% (firefighters) to 22% (psychiatric nurses), and was associated with psychological strain and suicidal ideation. The SEM showed that dysfunctional coping strategies, self-efficacy, previously experienced and work-related trauma, years on job, and female sex explained up to 78% of PTSS and that PTSS itself explained up to 68% of the psychological strain experienced in the different professions. Independent of the profession, dysfunctional coping such as alcohol use, avoidance, and distraction, as well as work-related trauma were the most robust predictors of PTSS. However, while self-efficacy was a risk factor for police officers, firefighters and ambulance personnel, it was a protective factor for emergency and psychiatric nurses. Furthermore, female sex was only a risk factor for ambulance personnel and emergency nurses. In agreement with prior research, emergency personnel and rescuers exhibited enhanced prevalence of PTSS and suspected PTSD, leading to significant psychological strain and suicidal ideation. However, risk factors varied across the professions. Thus, the development of profession-specific trainings to improve self-efficacy and coping with work-related stressors to reduce PTSS, and enhance quality of life, is needed for individuals in such high-risk professions.

**Clinical Trial Registration:** ClinicalTrials.gov Nr. NCT03842553.

## Introduction

Employees of rescue and emergency services are frequently subjected to traumatic experiences, and as the risk of posttraumatic stress disorder (PTSD) increases with the number of traumatic events experienced, professionals involved in rescue are at high risk of PTSD (1, 2). Rescue workers feature a higher prevalence of PTSD than does the general population (1, 3). However, PTSD prevalence across different occupational groups, including firefighters, police officers, ambulance personnel, and other rescue teams varies as widely as from 0 to 46% (4, 5). Profession-specific analyses support previous findings that ambulance personnel exhibit higher prevalence rates of PTSD than do firefighters, rescue teams, and police officers (1, 6). Similar professions, such as emergency and psychiatric nurses, have a comparable high risk of trauma exposure and thus show higher prevalence rates of PTSD (7).

Several meta-analyses have identified critical risk factors across professions: sex, previous trauma, number of work-related traumatic events, lack of social support, and dysfunctional coping strategies (8, 9). On the other hand, resilience, a sense of coherence, and self-efficacy can be protective factors (3, 9–12). While some variables cannot be changed, training and coaching can actively influence coping strategies and self-efficacy. Coping encompasses cognitive and behavioral strategies employed by an individual to manage a stressful situation and the attendant negative emotions and can thus determine whether a stressful situation leads to the development of posttraumatic stress symptoms (PTSS) and depression or to the continuation of a normal or even a better life (13–15).

Hence, to inform effective interventions, much research has focused on elucidating factors influencing the development of PTSD symptoms among rescue workers (16); special attention was given to those involved in disasters [i.e., earthquakes, floods, war, terror attacks, or airline disasters (5, 17–21)] or professionals who are repeatedly confronted with traumatic events during work, such as emergency personnel, ambulance personnel, and police officers. Such investigations showed that the most salient predictors of PTSD symptoms in rescue workers include previous work-unrelated traumas, number of work-related traumatic events, the female sex, self-efficacy, and coping strategies. Even though personnel of rescue and emergency services are often engaged in the same type of emergency, they vary regarding the tasks and responsibilities and receive different training in coping with traumatic events and stress. Thus, the salience of risk factors for the development of PTSS might vary across the different professions.

Through the present cross-sectional survey, we aimed to examine how these risk factors are related to PTSS and well-being across different professions of rescue workers using regression and structural equation modeling. We expected high self-efficacy and problem-focused coping strategies to be protective factors; and dysfunctional coping strategies, such as substance use and avoidance behavior, as well as female sex to be risk factors for the development of PTSS across all professions. We further hypothesize that the prevalence of suspected PTSD varies across the investigated professions, but that independent of the profession PTSS are associated with reduced well-being and suicidal ideation.

## Materials and Methods

### Participants

In the year 2015 we collected data from 1002 rescue workers in the canton Bern, Switzerland. The participation in the anonymous online study was voluntary and promoted by the department heads at the places of work corresponding to the studied professions. The study was deemed exempt from human subjects review as the survey was voluntary and anonymous and participants gave consent to use the data with their participation. The sample consisted of 499 (18% female) police officers from the cantonal police forces, 239 (4% female) firefighters from the professional fire service, 97 (43% female) ambulance personnel, 85 (86% female) emergency staff of the University Hospital of Bern, and 82 (68% female) qualified staff of two acute care wards of the University Hospital of Psychiatry in Bern. Of all participants, 61% were married or in a stable relationship, 31% were single, and 8% were divorced. The mean age of the study subjects was 39.6 (SD, 9.6; range, 21–66). Participants reported an average job experience of 14.7 years (SD, 9.7).

Missing data was in the following variables: self-efficacy 1.4%, subscale “Positive Distress Index” (BSI PSDI) of the brief symptom check list (BSI) 11.7% (only used to describe sample in Table 1) and suicidal ideation 0.6%.

**Table 1:** Demographic and clinical characteristics.

	**Police**	**Fire service**	**Ambulance service**	**Emergency staff**	**Psychiatric nurses**
Group size	*n* = 499	*n* = 239	*n* = 97	*n* = 85	*n* = 82
Mean age (SD)	38.4 (9.6)	42.3 (8.2)	38.7 (10.0)	38.7 (9.6)	40.9 (11.6)
% female	18%	4%	43%	86%	68%
Mean work experience in years (SD)	12.9 (9.6)	18.4 (8.4)	12.0 (9.0)	16.0 (9.6)	17.1 (11.0)
Living in a relationship	59%	74%	62%	51%	48%
Median PTSS (IQR)	5 (7.0)	3 (6.0)	6 (8.0)	7 (7.0)	7 (7.8)
Suspected PTSD	15%	8%	15%	18%	22%
Trauma before	31%	24%	37%	49%	54%
One or more trauma(s) during work	87%	81%	93%	99%	91%
Median BSI GSI (IQR)	0.20 (0.32)	0.12 (0.26)	0.18 (0.35)	0.22 (0.27)	0.22 (0.37)
Median BSI PST (IQR)	8.0 (11.0)	5.0 (9.0)	7.0 (14.0)	8.0 (11.0)	9.0 (11.5)
Median BSI PSDI (IQR)	1.30 (0.49)	1.23 (0.50)	1.23 (0.54)	1.30 (0.50)	1.25 (0.50)
Median GHQ (IQR)	8.0 (6.0)	7.0 (3.0)	8.0 (6.0)	8.0 (4.0)	9.0 (8.0)
PSES	29.0 (3.0)	29.0 (5.0)	30.0 (4.0)	30.0 (3.0)	31.0 (4.0)
Suicidal ideation	4.6%	2.5%	6.2%	3.5%	14.6%
Work-related conditions	1.60 (1.00)	1.67 (1.00)	1.80 (1.00)	2.00 (0.90)	2.20 (1.05)
Situations including people	2.00 (1.00)	1.88 (1.00)	2.00 (0.75)	2.25 (0.62)	2.23 (1.05)

*PTSS, Posttraumatic Symptom Scale; BSI GSI, Brief Symptom Inventory, Subscale Global Severity Index; BSI PST, Brief Symptom Inventory, Subscale Positive Symptom Total; BSI PSDI, Brief Symptom Inventory, Subscale Positive Distress Index; GHQ, General Health Questionnaire 12; PSES, General Perceived Self-Efficacy Scale*.

### Materials

Participants completed a 20-min online questionnaire regarding socio-demographic information, number of traumatic events before and during the course of their work, years on the job, coping with stressful and traumatic events, the burden of stressful job-related circumstances, self-efficacy, general psychological strain, posttraumatic stress symptoms, suicidal ideation, and well-being.

*Self-efficacy* was measured using the General Perceived Self-Efficacy Scale (22), a 10-item self-report instrument for personality diagnostics based on Banduras's concept of perceived self-efficacy that measures the general optimistic competence expectation (23).

The usefulness of different *Coping Strategies* was rated on 5-point Likert scale, ranging from a score of 1, indicating “not helpful;” to 5, “very helpful.” According to the operationalization of Coolidge (24), coping strategies were divided into three main subscales: (I) *problem-focused coping strategies*, which was comprised of the subscales of “instrumental support” (getting help and advice from psychologic professionals) and “active job coping” (debriefing with operation team, supervisor, and work colleagues); (II) dysfunctional coping strategies, which included “substance use” (alcohol or medication), “avoidance” (avoidance of situations and emotions associated with the stressful/traumatic situation), and “self-distraction” (turning to work or other activities to take the mind off the stressful situation); (III) emotion-focused coping strategy (humor, getting emotional support from close friends, thinking about the positive aspects of the job).

The burden of *stressful job-related circumstances*, such as dealing with *situations including people* (aggressive and violent people, dealing with deaths or suicide, threats, dealing with relatives, involvement of children) and *job-related conditions* (incorrect or wrong information about the emergency situation, shift work, time pressure). Participants stated if they had ever experienced a presented situation, and if so, whether the situation caused a light or heavy stress burden.

*Posttraumatic stress symptoms* were assessed with the German Version of the Post Traumatic Symptom Scale (PTSS-10) (25, 26), a self-rating scale used in screening and follow-up studies on catastrophe that measures the most common posttraumatic symptoms (sleep disorders, nightmares, fear of reminders, etc.) (27, 28). Attained values above 12.5 were associated with a suspected diagnosis of PTSD (29).

*Mental well-being* was assessed using the German version of the shortest General Health Questionnaire 12 (GHQ-12) (30, 31): originally a 60-item self-rating measurement developed to measure current mental well-being (consisting of two subscales: psychological and physiological well-being), it is widely used to detect psychological strain in the general population.

*Psychological distress and psychiatric disorders* were assessed with the German Version of the Brief Symptom Inventory (BSI). The 53-item self-report questionnaire measures symptoms of general psychopathology. Relevant to the current study are the three global scales that evaluate global psychological distress: the Global Severity Index (GSI), the Positive Distress Index (PSDI), and the Positive Symptom Total (PST). Additionally, participants were asked if they had *suicidal ideation* in the 12 months prior to the questionnaire.

### Statistical Analyses

**Figure 1:**
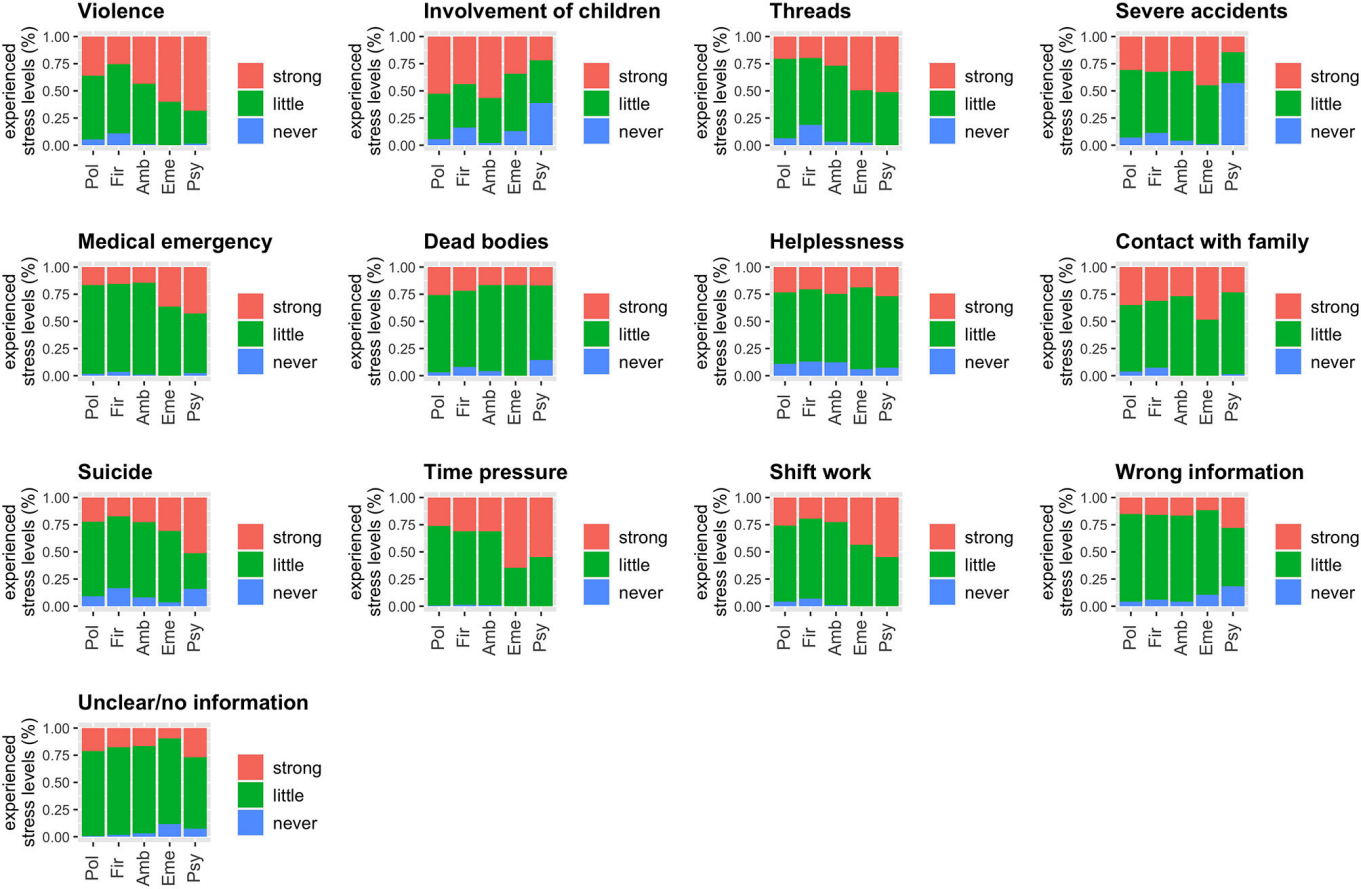
Stressful job-related circumstances, such as dealing with situations involving people (aggressive and violent people, dealing with deaths or suicide, threats, dealing with relatives, involvement of children) and job-related conditions (incorrect or wrong information about the emergency situation, shift work, time pressure) are rated regarding experienced stress levels (never = never experienced; little = no or little stress; strong = strong or very strong stress).

Differences in PTSS scores among groups were tested with a one-way ANCOVA, with work experience, age, and sex as covariates. Proportions of participants with a suspected diagnosis of PTSD were compared with a five-sample test for equality of proportions without continuity correction. For multiple regression, we investigated multicollinearity of the regressors beforehand, which all had a variance inflation factor (VIF) of <4; a cutoff of five is typically suggested (32). We used multiple regression as an exploratory approach to subset relevant variables that featured a significant relationship with PTSS in order to create a global causal model of PTSS symptoms, which could then be tested with structural equation modeling (SEM) for each group; the final model tested is shown in Figure 1. Two latent variables were considered in our model: “dysfunctional coping” has been regressed on “substance use,” “avoidance” and “self-distraction,” and “psychological strain” on the two global scales of the BSCL (GSI, PST) and two GHQ (psychological and physiological well-being) measures. To assess model fit, the degree to which SEM fit the sample data, we used the chi-square test, the *R*^2^, the comparative fit index (CFI), the Tucker-Lewis index (TLI), and the root mean square error of approximation (RMSEA). All analyses were performed with R v4.0.2 (33). For structural equation modeling, we used lavaan v.0.6-7 (34). The STROBE guidelines were used to ensure the reporting of this observational cross-sectional study (35).

### Software and Data Availability

STROBE checklist and the analysis pipeline to fully reproduce the results, tables, and figures of this paper are available at osf.io/c7xj2. A subset of the raw data to reproduce the main results of this paper will be online during or after publication.

## Results

### Posttraumatic Symptoms Among the Considered Professions

The comparison of PTSS scores among the occupational groups demonstrated that individuals working as emergency-room staff or in psychiatry services featured the highest PTSS (median = 7 for both), while working at the fire service was associated with the lowest PTSS (median = 3); the difference between groups was significant (*F*_4, 994_ = 6.21, *p* < 0.0001). The covariate sex was also significant; females were associated with an increase in PTSS (*F*_1, 994_ = 10.7, *p* = 0.001), while age and years of work experience were non-significant (age: *F*_1, 994_ = 2.63, *p* = 0.11; years of work experience: *F*_1, 944_ = 1.41, *p* = 0.24). The five occupational groups differed significantly regarding the prevalence of suspected PTSD: individuals employed in emergency (18%) or psychiatry services (22%) had the highest proportions of suspected PTSD, followed next by those in ambulance (15%) and police services (15%), and lastly by individuals in the fire service (8%) (*X*^*2*^ = 14.0, df = 4, *p* = 0.007; Table 1). The occurrence of suicidal ideation was also significantly different among groups (*X*^*2*^ = 20.0, df=4, *p* = 0.0005): psychiatric nurses exhibited the highest rates followed by those employed in ambulance and police services (Table 1). Further, the burden of *stressful job-related circumstances*, such as dealing with *situations including people* (e.g., aggressive and violent people, dealing with deaths or suicide) (*F*_4,997_ = 6.59, *p* < 0.0001) and *work-related conditions* (e.g., shift work, time pressure) (*F*_4,997_ = 14.7, *p* < 0.0001) significantly differed among occupational groups as (Figure 1).

### Regression Model

We created a multiple regression model to find variables strongly associated with PTSS (*F*_20,961_= 23.9; *p* < 0.0001) and explained 32% of the variance of PTSS (adjusted *R*^2^). Dysfunctional coping (substance use, avoidance, self-distraction) and suicidal ideation exhibited the strongest relationship (Table 2). Furthermore, the female sex, work experience in years, work-related trauma, previous work-unrelated trauma, and the professions involving psychiatry and emergency were positively associated with PTSS. However, self-efficacy was only protective for the psychiatric and emergency staff (significant interaction), suggesting a negative association with PTSS (slope coefficients, associated *t*-scores, and *p*-values are shown in Table 2).

**Table 2:** Regression estimates in a model of PTSS scores as outcomes and 13 predictor variables with three interaction terms sorted according to their relative importance as indicated by the *t*-score.

	**Estimate**	**95% CI**	***t*-score**	***p*-value**
*(Intercept)*	−10.98	From −15.88 to	−4.40	<0.0001
		−6.09		
Coping: substance use	2.69	From 2.07 to 3.31	8.47	<0.0001
Suicidal ideation (Yes)	4.42	From 3.13 to 5.71	6.73	<0.0001
Coping: avoidance	1.00	From 0.59 to 1.42	4.76	<0.0001
Coping: self-distraction	0.75	From 0.40 to 1.10	4.20	<0.0001
Sex (female)	1.49	From 0.74 to 2.24	3.92	<0.0001
Work experience (in years)	0.058	From 0.03 to 0.09	3.74	0.0002
Trauma work-related (yes)	1.61	From 0.76 to 2.47	3.70	0.0002
Profession: Emergency	16.05	From 6.43 to 25.68	3.27	0.001
Emergency^*^Self-efficacy	−0.52	From −0.84 to −0.20	−3.18	0.002
Self-efficacy	0.24	From 0.09 to 0.39	3.17	0.002
Trauma work-unrelated	0.84	From 0.25 to 1.43	2.81	0.005
Psychiatry^*^Self-efficacy	−0.48	From −0.82 to −0.14	−2.76	0.006
Profession: Psychiatry	14.12	From 3.47 to 24.76	2.60	0.009
Coping: active	−0.43	From −0.85 to −0.01	−1.99	0.047
Coping: instrumental support	0.34	From −0.02 to 0.69	1.84	0.066
Coping: emotional focused	−0.19	From −0.62 to 0.24	−0.87	0.39
Ambulance service^*^Self-efficacy	0.094	From −0.20 to 0.39	0.63	0.53
Profession: Police	0.95	From −4.49 to 6.39	0.34	0.73
Profession: Ambulance service	−1.18	From −9.70 to 7.33	−0.27	0.78
Police^*^Self-efficacy	−0.0013	From −0.19 to 0.19	−0.01	0.99

In a second model, we also included two additional variables regarding the burden of stressful job-related circumstances and compared the two model fits with an ANOVA (*F*_10,951_ = 0.92, *p* = 0.51). The models did not differ significantly, and the Akaike Information Criterion (AIC) increased after adding these two variables (AIC: 5659; larger model: 5669). These findings indicated the superiority of the original, simpler model that lacked the additional variables.

### Structural Equation Model

**Figure 2:**
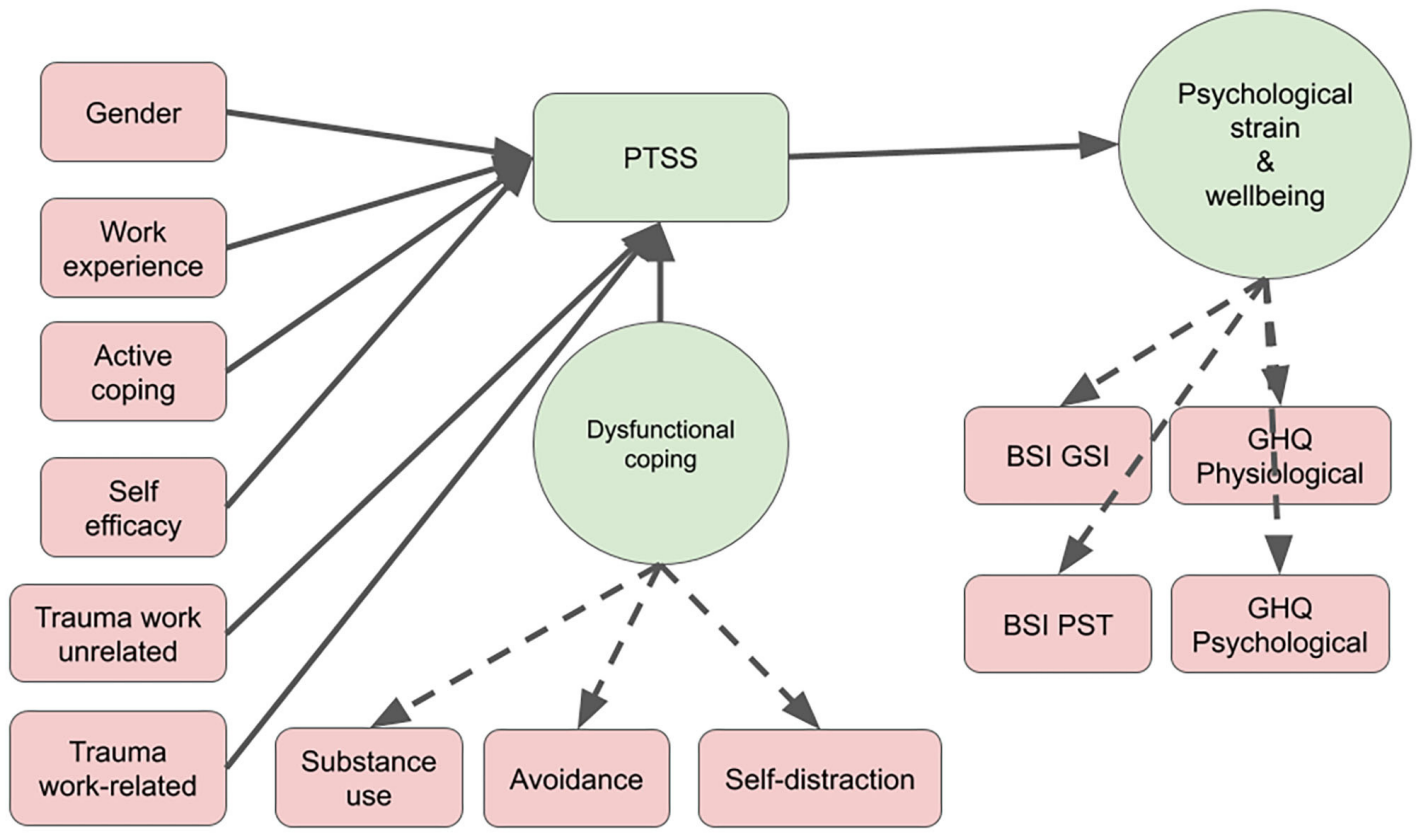
Structural equational modeling: variables associated with PTSS and psychological strain. PTSS, Posttraumatic Symptom Scale; BSI GSI, Brief Symptom Inventory, Subscale Global Severity Index; BSI PST, Brief Symptom Inventory, Subscale Positive Symptom Total; GHQ, General Health Questionnaire 12, subscales psychological and physiological well-being.

We fitted a causal model based on the significant regressors from the previous analysis to explain PTSS and psychological strain (Figure 2; for detailed results of chi-square and fit indices, see Supplementary Text 1). This model explained 37–78% of the variance (*R*^2^) of PTSS for the different groups and 48–68% of the variance of psychological strain (Table 3).

**Table 3:** Structural equation model regressions: path coefficients for explanatory variables for the five occupational groups.

**Group**	**Response variable**	**Explanatory variables**	**Coefficient**	**95% CI**	***p-value***
	**(variance explained *R^**2**^*)**				
Police	PTSS (0.48)	Dysf. coping	0.81	From 0.39 to 1.23	0.0002
		Active coping	0.01	From −0.16 to 0.18	0.89
		SE	0.26	From 0.05 to 0.46	0.013
		Trauma before	0.14	From −0.04 to 0.33	0.12
		Trauma work	0.19	From −0.00 to 0.38	0.054
		Work experience	0.07	From −0.11 to 0.25	0.42
		Female	0.04	From −0.14 to 0.22	0.66
	Psychological strain (0.68)	PTSS	0.82	From 0.68 to 0.96	<0.0001
Fire service	PTSS (0.42)	Dysf. coping	0.59	From 0.39 to 0.79	<0.0001
		Active coping	−0.03	From −0.14 to 0.08	0.57
		SE	0.23	From 0.11 to 0.34	<0.0001
		Trauma before	−0.01	From −0.12 to 0.11	0.93
		Trauma work	0.13	From 0.01 to 0.24	0.032
		Work experience	0.09	From −0.02 to 0.21	0.12
		Female	−0.03	From −0.15 to 0.08	0.55
	Psychological strain (0.57)	PTSS	0.76	From 0.69 to 0.83	<0.0001
Ambulance service	PTSS (0.78)	Dysf. coping	0.63	From 0.53 to 0.72	<0.0001
		Active coping	−0.08	From −0.16 to −0.01	0.035
		SE	0.15	From 0.07 to 0.23	<0.0001
		Trauma before	0.12	From 0.05 to 0.20	0.001
		Trauma work	0.11	From 0.03 to 0.19	0.007
		Work experience	0.06	From −0.03 to 0.14	0.17
		Female	0.15	From 0.07 to 0.22	0.0001
	Psychological strain (0.67)	PTSS	0.82	From 0.78 to 0.86	<0.0001
Emergency staff	PTSS (0.37)	Dysf. coping	0.38	From 0.14 to 0.62	0.002
		Active coping	−0.04	From −0.23 to 0.15	0.68
		SE	−0.20	From −0.39 to −0.02	0.027
		Trauma before	−0.10	From −0.28 to 0.08	0.28
		Trauma work	0.20	From 0.02 to 0.37	0.028
		Work experience	0.27	From 0.09 to 0.44	0.003
		Female	0.26	From 0.08 to 0.43	0.004
	Psychological strain (0.48)	PTSS	0.69	From 0.58 to 0.80	<0.0001
Psychiatric nurses	PTSS (0.41)	Dysf. coping	0.45	From 0.16 to 0.74	0.002
		Active coping	0.10	From −0.11 to 0.31	0.35
		SE	−0.35	From −0.54 to −0.15	0.0005
		Trauma before	0.17	From −0.04 to 0.38	0.12
		Trauma work	0.08	From −0.13 to 0.29	0.44
		Work experience	0.23	From 0.03 to 0.43	0.021
		Female	0.01	From −0.20 to 0.23	0.89
	Psychological strain (0.65)	PTSS	0.81	From 0.72 to 0.89	<0.0001

*Dysf. Coping, dysfunctional coping; PTSS, Posttraumatic Symptom Scale; SE, Self-Efficacy Scale; Psychological Strain, latent variable including Brief Symptom Inventory (subscales: Global Severity Index, Positive Symptom Total) and General Health Questionnaire (subscales: physiological well-being; psychological well-being). Dependent variables (italics) are in rows containing the explained variance (R^2^)*.

We found that dysfunctional coping featured significant positive-paths coefficients in all groups (Table 3). For active coping, we found a significant but small effect in relation to the ambulance service. Work-related trauma significantly predicted symptoms in all groups except psychiatry. Years of work experience was associated with a PTSS-symptom increase for emergency and psychiatric nurses. PTSS demonstrated a significant influence on psychological strain across all groups. High levels of self-efficacy were negatively correlated with PTSS among psychiatric and emergency staff, while they were positively correlated with PTSS in the ambulance, police, and fire services. The female sex had a significant positive association with PTSS symptoms only for the emergency and ambulance services (factor loadings of the latent variables are shown in Table 3).

## Discussion

Rescue workers are regularly engaged in traumatic events and are at high risk of PTSS. Though rescue workers of different professions are often engaged in the same type of emergency, they have different tasks and responsibilities and receive different training in coping with traumatic events and stress; hence, we speculated that the salience of risk factors for PTSS vary across their respective professions. To our knowledge, this is the first cross-sectional survey investigating how these risk factors are related to PTSS and well-being across 1,000 emergency personnel and rescue workers of five different professions and settings within the Bern metropolitan area (Switzerland). We further compared the prevalence rates of suspected PTSD across the investigated professions. We used a regression analysis to identify relevant factors that influence PTSS to construct and test a causal model using structural equation modeling, which tested how well these factors explain the outcome variables of PTSS and psychological strain.

The prevalence rate for suspected PTSD of the investigated professions mostly agree with the findings of previous research: firefighters featured the lowest rates of PTSD (8%), followed by professionals in the police and ambulance services (15%), emergency staff (18%), and psychiatric nurses (22%) (1, 7, 32). According to the results of the regression analysis, working in an emergency or psychiatry unit significantly increases the risk of PTSS. Our findings concerning emergency personnel and intensive care unit nurses supports the report of 20–29% of professionals in these fields meeting PTSD symptom criteria (7, 36). The repeated exposure of these personnel to traumatic situations, stressful events, and time pressure may explain the high prevalence of suspected PTSD in this profession. In addition, While other occupational groups such as police and firefighters are prepared during their training for the risk of developing PTSS and learn possible intervention to improve mental health, such training modules are often lacking in the nursing profession.

Studies on the prevalence rates of PTSD among psychiatric nurses showed much lower rates than those of suspected PTSD (37–39) found by the present study: 5–14 and 22%, respectively (32, 36, 38). A dearth of training in coping with the stress of exposure to assault, the potential for assault, and inpatient suicide, as well as the lack of routine (39), structured debriefing meetings after such critical events to promote resilience may account for the results (32, 36, 38). Our data further indicated that psychiatric nurses experienced significantly higher levels of stress when confronted with suicide than did other rescue workers (Figure 1). Feelings of worthlessness associated with feeling of inability to prevent the patients' death may underlie this significantly greater load of mental stress (37, 39).

The observed prevalence rates of suspected PTSD among rescuer workers outside of hospitals supports previous reports of the highest being among ambulance personnel, followed by police officers and firefighters. The fact that ambulance personnel are exposed to greater pressure and stress, due to more emergency calls and close contact with the victims, might account for this finding (1, 37). The difference highlights the heterogeneity among the various professions, even when the different types of rescue workers and emergency personnel are sometimes deployed to the same emergency. This variance might further be explained by work-specific circumstances, psychological preparedness, training, gender differences and personality traits.

The results of the SEM showed that the female sex, previously experienced work-unrelated trauma, work-related trauma, years on the job, dysfunctional coping strategies, problem-focused coping strategies, and self-efficacy explained between 37 and 78% of the PTSS, while PTSS itself explained between 48 and 68% of the psychological strain across the different professions. However, dysfunctional coping strategies, experienced work-related trauma, and self-efficacy were the most robust and strongest predictors for PTSS across most professions. A large body of previous research has reported an increase in the use of alcohol as a coping strategy to forget traumatic events in individuals exposed to disasters (40–42). Conrod and Stewart (42) suggest that PTSD and alcohol use/abuse appears to be related through a self-medication process. Thus, alcohol dampens the physiological stress response and emotional memory, and thus, has a short-term arousal- and anxiety-reducing effect, which is used for the management of PTSS, particularly for hyper-arousal and intrusion symptoms (43). However, in the long-term, alcohol use might impair the natural recovery process, preventing habituation to the traumatic experience and prolonging the intrusive PTSS and therefore maintain and intensify the PTSS symptomatology and increase the risk of alcohol dependence with all its serious medical consequences. Thus, preventative interventions should focus on assisting employees at a risk to develop more adaptive coping strategies for managing intrusive symptoms of hyper-arousal and re-experiencing such as relaxation trainings. However, even more importantly, employees should be informed about the severe consequences of dysfunctional coping strategies, such as alcohol use or avoidance, following exposure to traumatic events for the development of PTSS. Unexpectedly, problem-focused coping strategies, such as professional psychological support, debriefing in the team, talking with colleagues and supervisors seemed not to be as protective for PTSS, as reported in previous research.

Unexpectedly, self-efficacy showed a complex relationship across the different professions: while high levels were protective and associated with lower PTSS among psychiatric and emergency nurses, they were a risk factor among police officers, firefighters, and ambulance personnel. This latter finding may be accounted for reduced controllability and unpredictability of situations experienced by such rescue workers or gender specific differences. Another explanation might be an overestimation of ability: being too confident about one's capability of dealing with every situation and solving every problem alone might lead to higher psychological strain when confronted with unexpected and uncontrollable emotional distress and symptoms after traumatic events. Various studies investigating potential contributors to PTSS development but also posttraumatic recovery show that self-efficacy emerges as a central mediator for the development and recovery of PTSS (44–48). Self-efficacy is the belief in personal competence to deal with stressful situations and the capability to better regulate the own functioning. Thus, high levels of self-efficacy seem to buffer against the adverse effects of trauma and foster resilience (48). According to Chung et al. (48), self-efficacy is an enabling and protective process, which helps people to control their thinking, and to regulate their emotions, feelings, and affects. Low levels of self-efficacy are therefore associated with the inability to control one's thoughts, difficulties in regulating emotions, increased anxiety and distress leading to dysfunctional coping strategies. Lasting PTSS may negatively influence self-efficacy and the belief in personal competence to deal with stressful situations highlighting the importance of long-term follow-up of individuals recently exposed to trauma. However, in the present survey the information about the time point of the experienced trauma is missing, and therefore, difficult to interpret.

The other variables such as female sex, years on job, work-related and work-unrelated trauma were significant predictors in the overall regression analysis but varied in their significance in the SEM, further highlighting the heterogeneity across the different professions (for a detailed discussion of these variables, see Supplementary Text 2). This finding indicates that vocational training, professional assignment, environment, and possibly personality traits differ among the personnel of the different professions.

Apart from unchangeable factors such as sex, previous trauma, and years on job, our results show that dysfunctional coping strategies as well as self-efficacy most strongly predict PTSS across different professions engaged in emergencies. Resilience can be acquired and strengthened through psychotherapy and special training (49). With respect to such high-risk professions, strengthening resilience to improve coping with work-related stressors may reduce PTSS and enhance quality of life (49, 50). From this perspective, we have identified the following profession-specific areas for intervention. As our sample reveals a disproportionate number of female participants in the hospital staff group, the identified areas for intervention might not only be profession-specific but also gender-specific.

*Police officers and fire fighters*: Dysfunctional coping strategies and high self-efficacy are most strongly associated with PTSS, while work-related trauma is correlated with PTSS to a lesser extent.
*Ambulance personnel*: Dysfunctional coping strategies, high self-efficacy, and work-related trauma are risk factors for PTSS. Problem-focused coping strategies seem to be only marginally protective.
*Emergency nurses*: Dysfunctional coping strategies, years on the job, and work-related trauma are associated with PTSS, and high levels of self-efficacy are protective against PTSS.
*Psychiatric nurses*: Dysfunctional coping strategies and years on the job are risk factors for PTSS, while high levels of self-efficacy are protective against PTSS.

The urgency of improving these areas is underscored by our observation that 8–22% of the employees of the investigated professions develops a job-related psychiatric disorder, as well as the fact that PTSS is strongly associated with reduced psychological and physiological well-being and increases suicidal ideation (51). Suicide ideation is rather a consequence than a cause of PTSS, and there is extensive evidence that the presence, accessibility, and availability for firearms significantly increases the risk of suicide attempts (52–54). This needs very special attention for professions with armed forces, such as police but also the accessibility to medication in ambulance personnel, emergency and psychiatric nurses needs special attention.

The present study is subject to several limitations. The findings are based on retrospective self-report survey data, which should be replicated in prospective studies using more detailed interview methods—especially regarding PTSD symptomatology. Furthermore, the voluntary anonymous online survey, entitled “traumatization in emergency service,” might have addressed people that were already vulnerable or sensitized to the topic and consequently have caused a population bias. The investigated sample is not fully representative, but additional analysis of the percentage of participation within the investigated professions was between 20 and 66% (see Supplementary Table 1). Another critical point is the disproportionate number of female participants in the hospital staff group, since there is evidence that female gender is a risk factor for the development of internalizing disorders. The assessment of the exact number of experienced traumatic events during work and additional information regarding the trauma itself (direct vs. witnessed) would have afforded further insight into distinctive features of responses to different types of traumatic situations. Finally, the sample sizes of the different professions vary significantly, rendering a direct comparison among the professions difficult.

Based on our data, we cannot state a causality between work-related trauma and PTSS for several reasons: (1) posttraumatic stress symptoms were generally assessed and not in relation to work-related trauma, (2) the number of work-related and work-unrelated trauma was assessed but the information about the time point of the experienced trauma is missing. Thus, PTSS might come from work-unrelated trauma experienced before or outside the current work. However, the findings of the present survey show, that work-related trauma is a stronger predictor for PTSS than work-unrelated trauma. As the risk of PTSD increases with the number of traumatic events experienced (independent of the context), we can assume that individuals that additionally experienced work-unrelated traumatic events are even at a higher risk during employment in rescue and emergency services.

Summarized, the investigated emergency personnel and rescue workers exhibited enhanced prevalence of PTSS and suspected PTSD. PTSS itself was highly associated with psychological strain and increased suicidal ideation underlying the great burden and loss of quality of life in this population.

## Data Availability Statement

The raw data supporting the conclusions of this article will be made available by the authors, without undue reservation.

## Ethics Statement

Ethical approval was not provided for this study on human participants because the survey was voluntary and anonymous and participants gave consent to use the data with their participation.

## Author Contributions

LS, TM, and SW designed the study. LS conducted the data acquisition. SS analyzed the data. LS and SS wrote first draft of the manuscript and had full access to all of the data in the study and take responsibility for the integrity of the data and the accuracy of the data analysis. All authors discussed the findings and edited the final manuscript.

## Conflict of Interest

The authors declare that the research was conducted in the absence of any commercial or financial relationships that could be construed as a potential conflict of interest.

## References

[1] Berger W Coutinho ESF Figueira I Marques-Portella C Luz MP Neylan TC. Rescuers at risk: a systematic review and meta-regression analysis of the worldwide current prevalence and correlates of PTSD in rescue workers. Soc Psychiatry Psychiatr Epidemiol. (2012) 47:1001–11. 10.1007/s00127-011-0408-2 21681455 PMC3974968

[2] Neuner F Schauer M Karunakara U Klaschik C Robert C Elbert T. Psychological trauma and evidence for enhanced vulnerability for posttraumatic stress disorder through previous trauma among West Nile refugees. BMC Psychiatry. (2004) 4:34. 10.1186/1471-244X-4-34 15504233 PMC529265

[3] Streb M Häller P Michael T. PTSD in paramedics: resilience and sense of coherence. Behav Cognit Psychotherapy. (2014) 42:452–63. 10.1017/S1352465813000337 23714206

[4] Morren M Yzermans CJ Van Nispen RM Wevers SJ. The health of volunteer firefighters three years after a technological disaster. J Occup Health. (2005) 47:523–32. 10.1539/joh.47.523 16369116

[5] Stewart SH Mitchell TL Wright KD Loba P. The relations of PTSD symptoms to alcohol use and coping drinking in volunteers who responded to the Swissair Flight 111 airline disaster. J Anxiety Disord. (2004) 18:51–68. 10.1016/j.janxdis.2003.07.006 14725868

[6] Marmar CR Weiss DS Metzler TJ Ronfeldt HM Foreman C. Stress responses of emergency services personnel to the Loma Prieta earthquake Interstate 880 freeway collapse and control traumatic incidents. J Trauma Stress. (1996) 9:63–85. 10.1002/jts.2490090107 8750452

[7] Laposa JM Alden LE. Posttraumatic stress disorder in the emergency room: exploration of a cognitive model. Behav Res Ther. (2003) 41:49–65. 10.1016/S0005-7967(01)00123-1 12488119

[8] Brewin CR Andrews B Valentine JD. Meta-analysis of risk factors for posttraumatic stress disorder in trauma-exposed adults. J Consult Clin Psychol. (2000) 68:748. 10.1037/0022-006X.68.5.748 11068961

[9] Brown SP Westbrook RA Challagalla G. Good cope, bad cope: adaptive and maladaptive coping strategies following a critical negative work event. J Appl Psychol. (2005) 90:792. 10.1037/0021-9010.90.4.792 16060796

[10] Stratta P Capanna C Dell'Osso L Carmassi C Patriarca S Di Emidio G Resilience and coping in trauma spectrum symptoms prediction: a structural equation modeling approach. Pers Individ Dif . (2015) 77:55–61. 10.1016/j.paid.2014.12.035

[11] Wittmann L Moergeli H Martin-Soelch C Znoj H Schnyder U. Comorbidity in posttraumatic stress disorder: a structural equation modelling approach. Compr Psychiatry. (2008) 49:430–40. 10.1016/j.comppsych.2008.02.004 18702929

[12] Martin-Soelch C Schnyder U. Resilience and vulnerability factors in response to stress. Front Psychiatry. (2019) 10:732. 10.3389/fpsyt.2019.00732 31749715 PMC6843065

[13] Folkman S Moskowitz JT. Coping: pitfalls and promise. Annu Rev Psychol. (2004) 55:745–74. 10.1146/annurev.psych.55.090902.141456 14744233

[14] Freeman D Fowler D. Routes to psychotic symptoms: trauma, anxiety and psychosis-like experiences. Psychiatry Res. (2009) 169:107–12. 10.1016/j.psychres.2008.07.009 19700201 PMC2748122

[15] Lazarus RS. From psychological stress to the emotions: a history of changing outlooks. Annu Rev Psychol. (1993) 44:1–21. 10.1146/annurev.ps.44.020193.000245 8434890

[16] Wagner D Heinrichs M Ehlert U. Prevalence of symptoms of posttraumatic stress disorder in German professional firefighters. Am J Psychiatry. (1998) 155:1727–32. 10.1176/ajp.155.12.1727 9842783

[17] Benotsch EG Brailey K Vasterling JJ Uddo M Constans JI Sutker PB. War zone stress, personal and environmental resources, and PTSD symptoms in Gulf War Veterans: a longitudinal perspective. J Abnorm Psychol. (2000) 109:205. 10.1037/0021-843X.109.2.205 10895558

[18] Maguen S Lucenko BA Reger MA Gahm GA Litz BT Seal KH. The impact of reported direct and indirect killing on mental health symptoms in Iraq war veterans. J Trauma Stress. (2010) 23:86–90. 10.1002/jts.20434 20104592

[19] North CS Tivis L McMillen JC Pfefferbaum B Spitznagel EL Cox J. Psychiatric disorders in rescue workers after the Oklahoma City bombing. Am J Psychiatry. (2002) 159:857–9. 10.1176/appi.ajp.159.5.857 11986143

[20] Orcutt HK Erickson DJ Wolfe J. The course of PTSD symptoms among Gulf War veterans: a growth mixture modeling approach. J Trauma Stress. (2004) 17:195–202. 10.1023/B:JOTS.0000029262.42865.c2 15253091

[21] Perrin MA DiGrande L Wheeler K Thorpe L Farfel M Brackbill R. Differences in PTSD prevalence and associated risk factors among World Trade Center disaster rescue and recovery workers. Am J Psychiatry. (2007) 164:1385–94. 10.1176/appi.ajp.2007.06101645 17728424

[22] Schwarzer R Jerusalem M Skalen zur Erfassung von Lehrer- und Schülermerkmalen: Dokumentation der psychometrischen Verfahren im Rahmen der Wissenschaftlichen Begleitung des Modellversuchs Selbstwirksame Schulen. Berlin: Freie Universität Berlin (1999).

[23] Bandura A. Self-efficacy: toward a unifying theory of behavioral change. Psychol Rev. (1977) 84:191–215. 10.1037/0033-295X.84.2.191 847061

[24] Coolidge FL Segal DL Hook JN Stewart S. Personality disorders and coping among anxious older adults. J Anxiety Disord. (2000) 14:157–72. 10.1016/S0887-6185(99)00046-8 10864383

[25] Maercker A Posttraumatische-Stress-Skala-10 (PTSS-10). Angstdiagnostik–Grundlagen und Testverfahren. Berlin; Heidelberg: Springer (2003). p. 401–3.

[26] Weisaeth L. Torture of a Norwegians ship's crew: the torture, stress reactions and psychiatric aftereffects. Acta Psychiatr Scand. (1989) 80:63–72. 10.1111/j.1600-0447.1989.tb05255.x 2624136

[27] Aardal-Eriksson E Eriksson TE Holm A-C Lundin T. Salivary cortisol and serum prolactin in relation to stress rating scales in a group of rescue workers. Biol Psychiatry. (1999) 46:850–5. 10.1016/S0006-3223(98)00381-3 10494455

[28] Holen A Sund A Weisaeth L The Alexander Kielland Disaster March 27th 1980: Psychological Reactions Among the Survivors. Oslo: Division of Disaster Psychiatri, University of Oslo (1983).

[29] Stoll C Kapfhammer H Rothenhäusler H Haller M Briegel J Schmidt M. Sensitivity and specificity of a screening test to document traumatic experiences and to diagnose post-traumatic stress disorder in ARDS patients after intensive care treatment. Intensive Care Med. (1999) 25:697–704. 10.1007/s001340050932 10470573

[30] Goldberg D General Health Questionnaire (GHQ-12). Windsor, UK: Nfer-Nelson (1992).

[31] Schrnitz N Kruse J Tress W. Psychometric properties of the General Health Questionnaire (GHQ-12) in a German primary care sample. Acta Psychiatr Scand. (1999) 100:462–8. 10.1111/j.1600-0447.1999.tb10898.x 10626926

[32] Laposa JM Alden LE Fullerton LM. Work stress and posttraumatic stress disorder in ED nurses/personnel (CE). J Emerg Nurs. (2003) 29:23–8. 10.1067/men.2003.7 12556825

[33] Team RC R: A Language and Environment for Statistical Computing. (2018). Available online at: http://cran.univ-paris1.fr/web/packages/dplR/vignettes/intro-dplR.pdf

[34] Rosseel Y Lavaan: an R package for structural equation modeling and more. Version 0.5–12 (BETA). J Stat Software. (2012) 48:1–36. 10.18637/jss.v048.i02

[35] Von Elm E Altman DG Egger M Pocock SJ Gøtzsche PC Vandenbroucke JP The Strengthening the Reporting of Observational Studies in Epidemiology (STROBE) statement: guidelines for reporting observational studies. Ann Int Med. (2007) 147:573–7. 10.7326/0003-4819-147-8-200710160-00010 17938396

[36] Mealer ML Shelton A Berg B Rothbaum B Moss M. Increased prevalence of post-traumatic stress disorder symptoms in critical care nurses. Am J Respir Crit Care Med. (2007) 175:693–7. 10.1164/rccm.200606-735OC 17185650

[37] Berger W Figueira I Maurat AM Bucassio EP Vieira I Jardim SR. Partial and full PTSD in Brazilian ambulance workers: prevalence and impact on health and on quality of life. J Trauma Stress. (2007) 20:637–42. 10.1002/jts.20242 17721969

[38] Jacobowitz W. PTSD in psychiatric nurses and other mental health providers: a review of the literature. Issues Ment Health Nurs. (2013) 34:787–95. 10.3109/01612840.2013.824053 24131410

[39] Takahashi C Chida F Nakamura H Akasaka H Yagi J Koeda A. The impact of inpatient suicide on psychiatric nurses and their need for support. BMC Psychiatry. (2011) 11:38. 10.1186/1471-244X-11-38 21385448 PMC3063822

[40] Vlahov D Galea S Resnick H Ahern J Boscarino JA Bucuvalas M. Increased use of cigarettes, alcohol, and marijuana among Manhattan, New York, residents after the September 11th terrorist attacks. Am J Epidemiol. (2002) 155:988–96. 10.1093/aje/155.11.988 12034577

[41] Pfefferbaum B Doughty DE. Increased alcohol use in a treatment sample of Oklahoma City bombing victims. Psychiatry. (2001) 64:296–303. 10.1521/psyc.64.4.296.18598 11822207

[42] Conrod PJ Stewart SH Experimental studies exploring functional relations between posttraumatic stress disorder and substance use disorder. Trauma Subst Abuse. (2003):57–71. 10.1037/10460-003

[43] Stewart SH. Alcohol abuse in individuals exposed to trauma: a critical review. Psychol Bull. (1996) 120:83. 10.1037/0033-2909.120.1.83 8711018

[44] Benight CC Bandura A. Social cognitive theory of posttraumatic recovery: the role of perceived self-efficacy. Behav Res Ther. (2004) 42:1129–48. 10.1016/j.brat.2003.08.008 15350854

[45] Benight CC Cieslak R Molton IR Johnson LE. Self-evaluative appraisals of coping capability and posttraumatic distress following motor vehicle accidents. J Consult Clin Psychol. (2008) 76:677. 10.1037/0022-006X.76.4.677 18665695

[46] Benight CC Harper ML. Coping self-efficacy perceptions as a mediator between acute stress response and long-term distress following natural disasters. J Trauma Stress. (2002) 15:177–86. 10.1023/A:1015295025950 12092909

[47] Heinrichs M Wagner D Schoch W Soravia LM Hellhammer DH Ehlert U. Predicting posttraumatic stress symptoms from pretraumatic risk factors: a 2-year prospective follow-up study in firefighters. Am J Psychiatry. (2005) 162:2276–86. 10.1176/appi.ajp.162.12.2276 16330591

[48] Chung MC Allen RD Dennis I. The impact of self-efficacy, alexithymia and multiple traumas on posttraumatic stress disorder and psychiatric co-morbidity following epileptic seizures: a moderated mediation analysis. Psychiatry Res. (2013) 210:1033–41. 10.1016/j.psychres.2013.07.041 23978734

[49] Connor KM Davidson JR. Development of a new resilience scale: the Connor-Davidson resilience scale (CD-RISC). Depress Anxiety. (2003) 18:76–82. 10.1002/da.10113 12964174

[50] Martin-Soelch C Schnyder U Resilience and Vulnerability. (2020). 10.3389/fpsyt.2019.00732 PMC6843065 31749715

[51] Swanson JW McGinty EE Fazel S Mays VM. Mental illness and reduction of gun violence and suicide: bringing epidemiologic research to policy. Ann Epidemiol. (2015) 25:366–76. 10.1016/j.annepidem.2014.03.004 24861430 PMC4211925

[52] Weinberger SE Hoyt DB Lawrence HC Levin S Henley DE Alden ER Firearm-related injury and death in the United States: a call to action from 8 health professional organizations and the American Bar Association. Ann Int Med. (2015) 162:513–6. 10.7326/M15-0337 25706470

[53] Metzl JM MacLeish KT. Mental illness, mass shootings, and the politics of American firearms. Am J Public Health. (2015) 105:240–9. 10.2105/AJPH.2014.302242 25496006 PMC4318286

[54] Brent DA Perper JA Allman CJ Moritz GM Wartella ME Zelenak JP. The presence and accessibility of firearms in the homes of adolescent suicides: a case-control study. JAMA. (1991) 266:2989–95. 10.1001/jama.266.21.2989 1820470

