# Supplementary material for: Rescuers at Risk: Posttraumatic Stress Symptoms Among Police Officers, Fire Fighters, Ambulance Personnel, and Emergency and Psychiatric Nurses
Source: Front Psychiatry. 2021 Jan 19;11:602064. doi: 10.3389/fpsyt.2020.602064 (PMC7851799; doi:10.3389/fpsyt.2020.602064)
Supplement: Supplementary file 1 [file Data_Sheet_1.docx]

Supplementary Material

# Supplementary Results

## S1 Text: Structural Equation Model

The model was fitted for five groups and produced an overall chi-square of 913.1 with 305 degrees of freedom and the following chi-squares for each service: ambulance, *Χ*² = 111.0; fire, *Χ*² = 217.1; police, *Χ*² = 308.2; emergency, *Χ*² = 126.9; and psychiatry, *Χ*² = 149.9. This model explained 37%–78% of the variance (*R^2^*) of PTSS for the different groups and 48%–68% of the variance of psychological strain. The model had the following fit indices: comparative fit index, 0.879; Tucker-Lewis index, 0.849; and root mean square error of approximation, 0.100 (90% CI, 0.093–0.108). These values suggest an acceptable fit of the model.

For active coping, we only found a small, significant effect for the ambulance service (path coefficient: -0.08). Trauma at work also significantly predicted symptoms in all groups except psychiatry. Years of work experience was associated with a PTSS-symptom increase for emergency and psychiatric staff. Generally, PTSS demonstrated significant influence on psychological strain across all groups. Self-efficacy had a significant effect on symptoms in all the groups, with negative loadings of the path coefficient in the emergency and psychiatric staff and positive loadings in the other groups. The female sex had a significant positive association on PTSS symptoms only in the emergency and ambulance services. Factor loadings of the latent variables are shown in Table 4.

1. **Supplementary Discussion**

**S2 Text:**

*Previous work-unrelated trauma and work-related trauma:* Most of the rescue workers of the different professions reported experiencing one or more traumatic events during work (from 81% of firefighters to 99% of emergency staff), while there was a greater degree of heterogeneity regarding the experience of previous work-unrelated traumas across the professions (from 24% of firefighters to 54% of psychiatric nurses). In agreement with previous research, the regression analysis found that both variables were significant predictors for the development of PTSS across the investigated professions [1]. However, according to the structural equation model, previously experienced trauma seems to be relatively less important; work-related trauma, on the other hand, significantly predicted PTSS across all professions except emergency nurses. The association of work-related trauma with PTSS supports the findings of prior research. The personnel examined herein are at high risk of exposure to direct threats to their own wellbeing (e.g., threatened and actual assault) or indirect threats, such as involvement in events in which other persons are injured, threatened, or even killed, that present no direct danger to themselves [2-4]. However, based on our data, we are not able to distinguish between the two types of threats; elucidation of the differential of impacts [1, 5-8] that direct and indirect threats would help to identify distinctive features of response to different traumatic stressors. Regardless, special attention should be paid to work-related trauma and the development of training and debriefings after such events.

*Sex:* Another risk factor that could be confirmed by the present study is the female sex (2). There is evidence that female rescue workers exhibit a higher risk of developing PTSS relative to men (1,2). However, our profession-specific analysis showed that only women working in the ambulance and emergency services featured a higher risk for PTSS.

# Supplementary Tables

**S1 Table.** Approximate number of employees of the different professions investigated

|  | **Police** | **Fire service** | **Ambulance service** | **Emergency staff** | **Psychiatry staff** |
| --- | --- | --- | --- | --- | --- |
| Nr. of employees | 2500 | 360 | 160 | 115 | 300 |
| Nr. employees who participated in the survey | 499 | 239 | 97 | 85 | 82 |
| % participation | 19.96% | 66.38% | 60.63% | 73% | 27.33% |

% participation: Percentage of employees that participated in the online survey of each profession investigated. Nr.: number.

**REFERENCES**

1. Brewin, C.R., B. Andrews, and J.D. Valentine, *Meta-analysis of risk factors for posttraumatic stress disorder in trauma-exposed adults.* Journal of consulting and clinical psychology, 2000. **68**(5): p. 748.

2. Alden, L.E., M.J. Regambal, and J.M. Laposa, *The effects of direct versus witnessed threat on emergency department healthcare workers: Implications for PTSD Criterion A.* Journal of anxiety disorders, 2008. **22**(8): p. 1337-1346.

3. Laposa, J.M. and L.E. Alden, *Posttraumatic stress disorder in the emergency room: exploration of a cognitive model.* Behaviour research and therapy, 2003. **41**(1): p. 49-65.

4. Laposa, J.M., L.E. Alden, and L.M. Fullerton, *Work stress and posttraumatic stress disorder in ED nurses/personnel (CE).* Journal of emergency nursing, 2003. **29**(1): p. 23-28.

5. Berger, W., et al., *Rescuers at risk: a systematic review and meta-regression analysis of the worldwide current prevalence and correlates of PTSD in rescue workers.* Social psychiatry and psychiatric epidemiology, 2012. **47**(6): p. 1001-1011.

6. Marmar, C.R., et al., *Predictors of posttraumatic stress in police and other first responders.* Annals of the New York Academy of Sciences, 2006. **1071**(1): p. 1-18.

7. Marmar, C.R., et al., *Longitudinal course and predictors of continuing distress following critical incident exposure in emergency services personnel.* The Journal of nervous and mental disease, 1999. **187**(1): p. 15-22.

8. Martin, M., et al., *Predictors of the development of posttraumatic stress disorder among police officers.* Journal of Trauma & Dissociation, 2009. **10**(4): p. 451-468.
